# True prevalence of long COVID in children: a narrative review

**DOI:** 10.3389/fmicb.2023.1225952

**Published:** 2023-09-14

**Authors:** Susanna Esposito, Michela Deolmi, Greta Ramundo, Matteo Puntoni, Caterina Caminiti, Nicola Principi

**Affiliations:** ^1^Pediatric Clinic, Pietro Barilla Children’s Hospital, Department of Medicine and Surgery, University Hospital of Parma, Parma, Italy; ^2^Clinical and Epidemiological Research Unit, University Hospital of Parma, Parma, Italy; ^3^Università degli Studi di Milano, Milan, Italy

**Keywords:** COVID-19, COVID vaccines, long COVID, pediatric infectious diseases, SARS-CoV-2, viral variants

## Abstract

Contrary to what is true for adults, little is known about pediatric long COVID (LC). Studies enrolling children are relatively few and extremely heterogeneous. This does not allow to draw definitive conclusions on the frequency and pathogenesis of pediatric LC and limits the development of appropriate and effective measures to contain the clinical, social and economic impact of this condition on the pediatric population. Depending on the methods used to collect and analyze data, studies have found that the incidence rate of pediatric LC may vary from about 25% to less than 5%. However, despite true prevalence of pediatric LC cannot be exactly defined, studies comparing children with previous COVID-19 and uninfected controls have shown that most of the clinical manifestations detected in infected children, mainly mood symptoms, mental health disorders and heart abnormalities could be diagnosed with similar frequency and severity in uninfected subjects also. This seems to indicate that SARS-CoV-2 is the cause of pediatric LC only in a part of children and other factors play a relevant role in this regard. Pandemic itself with the persistent disruption of child lives may have caused persistent stress in all the pediatric population causing mood symptoms, mental health disorders or several organ and body system functional alterations, regardless SARS-CoV-2 infection. These suppositions suggest the need for long-term physical control of all the children after COVID-19 especially when they were already suffering from an underlying disease or have had a severe disease. Moreover, attention should be paid to the assessment of change in children’s emotional and behavioral functioning in order to assure adequate interventions for the best emotional and behavioral well being. However, whatever its origin, it seems highly likely that the prevalence of the pediatric LC is set to decline in the future. Preliminary observations seem to suggest that recently developed SARS-CoV-2 variants are associated with less severe COVID-19. This suggests that, as already seen in adults, a lower number of pediatric virus-associated LC cases should occur. Furthermore, the use of COVID-19 vaccines, reducing incidence and severity of SARS-CoV-2 infection, may reduce risk of LC development. Finally, elimination of restrictive measures should significantly reduce mood symptoms and mental health disorders.

## 1. Introduction

Monitoring of subjects previously infected by SARS-CoV-2 has shown that COVID-19 is not only an acute disease, generally solving in about 4 weeks, but that it can lead to long-term manifestations. Some patients develop persistent and often relapsing and remitting symptoms beyond some weeks after infection. This condition has been named as post-acute COVID-19 syndrome, post-acute sequelae of COVID-19 (PASC), post-COVID-19 condition and, more commonly, long COVID (LC) (Davis et al., 2023). In adults, to identify patients with LC and to allow studies aimed to define characteristics of this condition, several different definitions of LC have been prepared. The World Health Organization (WHO) has defined LC as a condition characterized by symptoms occurring 3 months from the onset of acute COVID-19 manifestations, last for at least 2 months and cannot be explained by an alternative diagnosis (World Health Organization, 2023c). The US National Institute of Health (NIH) has defined LC all the symptoms at least persisting or emerging 4 weeks after SARS-CoV-2 infection (National Institute of Health, 2023). Finally, the United Kingdom National Institute for Health and Care Excellence (NICE) has made a distinction between disease occurring from 4 to 12 weeks after infection and symptoms persisting beyond 12 weeks (National Institute for Health and Care Excellence (NICE), 2023). Despite these differences have led to problems in pooling results and in identifying LC characteristics, most of the studies have led to the conclusion that adult LC was a very common condition as up to 50% of people with previous SARS-CoV-2 infection could suffer from long-term clinical manifestations. More than 200 symptoms, involving different organ and body system, were associated with LC. Fatigue, malaise, altered smell and taste, breathlessness, and cognitive impairments were the most common, although neuropsychiatric, cardiovascular, pulmonary, hematological, gastrointestinal, renal, endocrine, dermatological, and musculoskeletal sequelae have been reported. Female sex, older age, comorbidities, the severity of acute disease, and obesity were the factors more frequently associated with LC development (Aiyegbusi et al., 2021; Groff et al., 2021; Michelen et al., 2021; Nasserie et al., 2021; Lopez-Leon et al., 2022; O'Mahoney et al., 2022). Moreover, it was stablished that, together with medical disorders, LC could cause relevant social and economic problems, for reduction of quality of life of the patient and his family, long absence periods off work, adjusted workloads, and loss of employment (Nittas et al., 2022).

Studies regarding development and characteristics of LC in children are few (Esposito et al., 2022; Fainardi et al., 2022). Most of them suffer from several limitations. First, the lack of a single criterion to identify children with LC. To perform studies in children, criteria prepared for adults were used. This led to enroll children with different characteristics, making it difficult to pool and evaluate the results. Only recently a specific definition of pediatric LC was prepared using robust consensus methodology (Stephenson et al., 2022). It is aligned to WHO definition (World Health Organization, 2023c) and can be used in the future to obtain more reliable data of pediatric LC characteristics. Moreover, pediatric LC studies were generally retrospective and very heterogeneous because of differences from design, site of recruitment, details of pre-existing diseases and severity of acute COVID-19, follow-up time, methods of LC symptom evaluation. In several cases, patients with self-reported symptoms without laboratory confirmed SARS-CoV-2 infection were enrolled (Ashkenazi-Hoffnung et al., 2021; Say et al., 2021; Buonsenso et al., 2022; Osmanov et al., 2022; Puntoni et al., 2023). Finally, several studies lacked a control group of viral test-negative children, limiting the ability to differentiate clinical manifestations directly attributable to SARS-CoV-2 infection from those ascribable to the potential damage of pandemic-induced behavior changes (Annam et al., 2022). As a consequence, several aspects of pediatric LC remain poorly defined. Among these, true prevalence and factors that can influence it. Knowledge of these aspects is essential to understand which is the true dimension of the problem and is the base for a detailed characterization of LC in children. Only knowing how many children suffer from LC and which factors may influence prevalence rates, adequate measures of prevention and treatment can be planned. In this narrative review, present knowledge on pediatric LC is discussed.

## 2. Methods

The MEDLINE/PubMed database was searched from 2020 to 30 April 2023 to collect the literature. The search included randomized placebo-controlled trials, controlled clinical trials, double-blind, randomized controlled studies, and systematic reviews and meta-analyses. Abstracts were excluded. The following combinations of keywords were used: “long COVID” OR “post-acute COVID-19” OR “post-COVID-19” AND “children” OR “adolescent” OR “pediatric” OR “paediatric” AND/OR “non-enveloped viruses.”

## 3. Presently available information on long COVID in children

All the studies that have evaluated long-term effects of SARS-CoV-2 infection in children have clearly evidenced that pediatric LC is a real disease, as it is in adults. However, pediatric LC prevalence varied significantly between studies. In most of the cases, it was shown that LC was very common, regardless of how this condition was defined. In the systematic review and meta-analysis by Lopez-Leon et al. including 21 retrospective studies with a total of 80,071 children, it was calculated that pediatric LC, defined according to the NIH definition, was 25.24% (95% confidence interval [CI], 18.17–33.02) (Lopez-Leon et al., 2022). Quite similar results were recently reported by Zheng et al. in a systematic review and meta-analysis of 40 retrospective studies with 12,424 children. In this case, criteria prepared by WHO to define LC were used (Zheng et al., 2023). It was shown that pediatric LC prevalence was 23.36% (95% CI, 15.27–32.53). However, the pooled prevalence of LC was closely related to the duration of the observation period following the acute illness. It was 26.41% (95% CI, 14.33–40.59) when the follow up was limited to 3–6 months, 20.64% (95% CI, 17.06–24.46) when it was extended to 6–12 months and 14.89% (95% CI, 6.09–26.51) when it lasted >12 months, showing the importance of the duration of follow-up in conditioning LC prevalence. High prevalence rates of LC in children were also reported by some prospective studies recently performed. Morello et al. enrolled 1,243 children with symptomatic or asymptomatic SARS-CoV-2 infection and assessed them at 3, 6, 12, and 18 months later. At 3 months post-onset, LC was diagnosed in 23% of the cases (Morello et al., 2023). Of these, about 50% remained symptomatic at 6 months, 12.9% at 12 months, and 5.1% at 18 months. Osmanov et al. monitored for several months a group of 518 children discharged from the hospital after COVID-19 and found that about 8 months later 126 of them (24.3%) had persistent symptoms (Asadi-Pooya et al., 2021).

Despite differences between studies, the most common manifestations were neurologic and psychiatric symptoms and cardiorespiratory symptoms. In particular, very common were mood symptoms (i.e., sadness, tension, anger, depression, and anxiety), fatigue, sleep disorders, altered taste and smell, headache, dyspnea, nasal congestion, chest pain, palpitation. In many children clinical manifestations involving different body organs and systems were evidenced (Lopez-Leon et al., 2022; Zheng et al., 2023). Several risk factors for LC development were identified, although those more frequently detected were older age, previous severe COVID-19 and pre-existing co-morbidities (Asadi-Pooya et al., 2021).

In a small number of studies, however, totally different conclusions were drawn. Very low prevalence rates of pediatric LC were found. A nationwide cross-sectional Danish study found persistent symptoms at 2 months after SARS-CoV-2 infection in 12.8% of children 0 to 3 years, in 4.4% of those 4 to 11 years, and in 4.7% of those aged 12–14 years (Kikkenborg Berg et al., 2022). Rao et al. reported that LC could be diagnosed in 3.7% of cases (Rao et al., 2022). Finally, a prevalence of about 2 and 0.8% was calculated by Radtke et al. (2021) and Borch et al. (2022).

The reason for these different findings can be ascribed to the heterogenicity of the studies that differed in criteria used to define LC, designs, settings, populations, follow-up time, symptom ascertainment methods, details of pre-existing comorbidities, and prior receipt of COVID-19 therapeutics and vaccines. Moreover, in most cases a high probability of bias was present. However, studies enrolling a control group, i.e., a group of children without SARS-CoV-2 infection, clearly showed that, despite global prevalence of signs and symptoms of disease suggesting LC was greater in patients with previous SARS-CoV-2 infection than in uninfected controls, most of these clinical manifestations, mainly mood symptoms, mental health disorders and heart functional alterations, were quite similar for frequency and severity in both groups. In the study by Kikkenborg Berg et al. comparison between infected and uninfected children revealed that signs and symptoms of LC could be detected in 40.0% versus 27.2% (odds ratio [OR], 1.78; 95% CI, 1.55–2.04, *p* < 0.0001) of children aged 0–3 years, 38.1% versus 33.7% (OR, 1.23; 95% CI, 1.15–1.31, *p* < 0.0001) of those 4–11 years, and 46.0% versus 41.3% (OR, 1.21; 95% CI, 1.11–1.32, *p* < 0.0001) in those 12–14 years (Kikkenborg Berg et al., 2022). Similarly, Rao et al. showed that at least 1 symptom of LC could be detected in 41.9% (95% CI, 41.4–42.4) of viral test–positive children and in 38.2% (95% CI, 38.1–38.4) of negative controls (Rao et al., 2022). Despite the accuracy of these results can be debated as criteria used to identify controls were different from study to study, and most of the studies had several relevant limitations (Dattner et al., 2021; Jarvis and Kelley, 2021; Radtke et al., 2021; Roge et al., 2021; Knoke et al., 2022), these findings strongly suggest that SARS-CoV-2 infection is not the only cause of pediatric LC and that, during pandemic, factors with a role as cause of children’s diseases. Were older age, female sex, severe acute-COVID-19, obesity, allergic disease and long-term health conditions (Asadi-Pooya et al., 2021; Miller et al., 2022; Molteni et al., 2022; Osmanov et al., 2022). In this regard, results of the prospective study by Morello et al. showed that age more than 10 years, prexisting conditions and hospitalization during the acute phase due to severity of disease increased the risk of developing LC of about 4, 2 and 3 times, respectively (Morello et al., 2023). Moreover, recent data indicate that persistent clinical manifestations after COVID-19 are more common in children with MIS-C. At this regard, Messiah et al. reported that 26.9% of children that have suffered from MIS-C reported chronic LC compared to 15.3% of those without Messiah et al. (2022).

Initially, it was supposed that many signs and symptoms of LC, including mental health disorders, digestive issues, heart disease, immune system problems, and muscular pain could be the consequence of the pathophysiological changes due to SARS-CoV-2 infection (Davis et al., 2023). Mitochondrial dysfunction, coagulopathy and endothelial damage, immune dysregulation commonly found in COVID-19 patients were reported as possible causes of these symptoms (Astin et al., 2023). However, a different explanation for symptoms development, applicable to both infected and uninfected subjects, could be suggested. It can be supposed that most of these symptoms were strictly dependent on the pandemic itself and the behavioral changes imposed on the entire population, including children, during pandemic. Several studies have shown that mitigation measures put in place by health authorities to reduce SARS-CoV-2 circulation (Ayouni et al., 2021) had significantly impacted on physical and mental health, education, and economic well-being of many children, of all ages, and in all countries (Samji et al., 2022; Solmi et al., 2022). Moreover, it was thought that anxious feelings of fear of COVID-19 for themselves, family members, relatives and friends had been significantly contributed to increase psychological and physical problems (Ayouni et al., 2021). It is now largely accepted that significant and protracted behavioral changes such as those occurred during pandemic could have caused the development of a chronic stress and that, in turn, this could have led to the clinical manifestations seen in most of the children regardless they were with or without infection (McEwen, 2017; Yaribeygi et al., 2017; Pavli et al., 2021; Agorastos and Chrousos, 2022; Bajoulvand et al., 2022; Davis et al., 2023). Chronic stress is associated with significant modifications of central nervous system structure and function that impact on memory, cognition and learning and the activity of several body organ and systems. Even the most common symptoms such as mood, fatigue, sleep disorders, orthostatic intolerance, decreased concentration, confusion, memory loss, exercise intolerance, hyperhidrosis, blurred vision, body temperature dysregulation, heart rate variability and palpitations, and dysphagia, are part of the clinical picture of dysautonomia, a condition that depends on a dysfunction of the sympathetic and/or parasympathetic autonomic nervous and has been found associated with stress (Gurel et al., 2022).

Due to the limitations of presently available data, further studies are needed to precisely quantify the role of SARS-COV-2 and pandemic-related factors as cause of diseases in children during pandemic. However, in panning new studies, it should be considered that new, previously unrecognized, factors, may significantly modify the prevalence of all the clinical manifestations emerged in children during pandemic regardless of whether or not they were related to SARS-CoV-2. Several variants of concern of the original SARS-CoV-2 have emerged and different transmissibility and virulence of each of them may significantly modify long-term disease prevalence (Tian et al., 2022). Highly effective vaccines have been licensed and this might have modified virus circulation and impact. Finally, restrictive measures have been progressively reduced or totally eliminated worldwide., especially after WHO declared COVID-19 over as a global health emergency with potential reduction of indirect effects of pandemic (World Health Organization, 2023a).

## 4. Variants and long COVID

Since the beginning of the COVID-19 pandemic, several variants of the wild-type SARS-CoV-2 have emerged. Some of them, particularly Alpha (B.1.1.7), Beta (B.1.351), Gamma (P1), Delta (B.1.617.2) and Omicron (B.1.1.529) variants, were defined as variants of concern (VoC), as, compared to the original strain, they showed different characteristics (World Health Organization, 2023b). Each of them was found to have its own transmissibility, virulence, resistance to acquired immunity from vaccines or previous infection, and ability to elude diagnostic and therapeutic measures. The most recent variants, Delta and Omicron, were designated VoC in May 2021 and in November 2021, respectively. The Delta variant was the dominant strain during summer and fall 2021 (Dhawan et al., 2022) and was characterized by a significant greater ability than the original strain and the previous variants to cause severe COVID-19 (Twohig et al., 2022). The Omicron variant, that became the cause of the largest wave of the pandemic and that with its subvariants, remains the leading cause of COVID-19, was found extremely transmissible and, in adults, generally associated with a lower risk of severe disease, hospitalization and death (Menni et al., 2022; Whitaker et al., 2022; Wrenn et al., 2022). As severity of COVID-19 is a risk factor for LC development, these findings suggested that a significant decline of LC prevalence should have occurred in adults in the last year when the Omicron variant was prevalent. The meta-analysis by Fernández-de-Las-Peñas et al. confirms this supposition (Fernández-de-las-Peñas et al., 2022). These authors analyzed 6 studies and 355, 512, 41.563, and 57.616 adult patients infected with the historical variant, the Alpha variant, the Delta variant, and the Omicron variant, respectively. Despite not marginal limitations of some of the studies, it was concluded that the available evidence indicated that Omicron patients could be at a lower risk of developing LC than those infected with other variants.

Data regarding role of variants as cause of severe COVID-19 in children resemble those collected in adults, although with same exceptions (Sumner et al., 2023). Butt et al. studied 985 propensity score-matched pairs of children with Delta and Omicron virus infection (Butt et al., 2022). Odds of moderate or severe/critical COVID-19 was significantly lower (adjusted odds ratio [AOR], 0.12; 95% CI, 0.07–0.18) in children with Omicron than in those with Delta. Jank et al. calculated the number of pediatric SARS-CoV-2 infections requiring COVID-19-related hospitalization or ICU admission in Germany during the whole pandemic period (Jank et al., 2023). They found that, apart from children <5 years, morbidity caused by SARS-CoV-2 infections among children in Germany decreased over the course of pandemic, reaching the lowest values during the period of Omicron prevalence. Compared to the Alpha period, the risk of hospitalization and ICU admission decreased during Delta (OR 0.60; 95% CI, 0.54–0.67 and OR 0.51; 95% CI, 0.42–0.61) and Omicron (OR 0.27; 95% CI, 0.24–0.30 and OR 0.06; 95% CI 0.05–0.08) Finally, data showing a reduced risk of severe infection with Omicron variant were reported by Bahl et al. with a study in which consecutive pediatric patients presenting to the emergency department with COVID-19 were included (Bahl et al., 2023). Incidence of severe disease in time intervals coinciding with Alpha, Delta, and Omicron variant predominance was evaluated. The odds of severe disease were significantly lower in the period of Omicron prevalence than in the period of Alpha prevalence (aOR 0.35; 95% CI, 0.21–0.60). All these findings should lead to conclude that, as for adults, the prevalence of LC in children should have progressively decreased with the evolution of the pandemic and the emergence of the Omicron variant. Unfortunately, pediatric data in this regard are few. However, the prospective study by Morello et al. seems to confirm what was expected (Morello et al., 2023). Studying the potential prognostic factors of LC development, these authors reported that regarding variants the ORs were 3.37 (95% CI, 1.81–6.26) for the original virus, 3.73 (95% CI, 2.32–6.00) for the Alpha variant, 1.18 (95% CI, 0.86–1.63) for the Delta variant and 0.47 (95% CI, 0.36–0.62) for the Omicron variant. All these findings seem to indicate that prevalence of pediatric LC depends on the type of the SARS-CoV-2 and that, at the moment, lower that previously reported LC could be expected (Morello et al., 2023).

## 5. Vaccines and long COVID

Several safe and effective vaccines against COVID-19 have been developed. They have significantly modified the course of pandemic, through the reduction in the number of new SARS-CoV-2 infections, COVID-19 cases, outpatient visits, emergency department admissions, hospitalizations and deaths (Watson et al., 2022). Medical, social, and economic advantages of vaccination were enormous. However, whereas the impact of vaccines on COVID-19 has been clearly evidenced, little is known on the effect of vaccines on LC prevalence. Theoretically, COVID-19 vaccines may be effective against LC both when administered before SARS-CoV-2 infection and when given after infection has already occurred or LC has already developed. Some answers to the question whether COVD-19 vaccines can somehow interfere with the development and course of LC can be given by the study by Byambasuren et al. (2023). These authors reviewed a total of 16 studies enrolling a total of 614,392 patients. In 12 studies impact of vaccines given before infection was evaluated. In 10 of them, a protective effect of immunization was shown, although protection increased with the increase in the number of administered vaccine doses. The OR of developing LC ranged from 0.22 to 1.03 after one vaccine dose, from 0.25 to 1 after two vaccine doses, and 0.16 after three vaccine doses. Positive results were also evidenced in the few studies aimed to evaluate impact on vaccination after infection. Risk of LC development was reduced (OR ranged from 0.38 to 0.91; Byambasuren et al., 2023). Further information on COVID-19 vaccine impact on adult LC was collected by Tran et al. who studied vaccines efficacy on symptoms of with LC in 455 immunized patients and 455 unvaccinated controls. By 120 days, a very mild reduction in the number of LC symptoms in immunized subjects was shown (mean difference −1.8; 95% CI, −3.0 to −0.5; Tran et al., 2023). Moreover, the rate of patients in remission was doubled (16.6% vs. 7.5%; Tran et al., 2023). However, all these studies had significant methodological limitations, a potential positive effect of COVID-19 vaccines, especially when given before SARS-CoV-2 infection, seems to have to be expected from the ever-increasing diffusion of the anti-COVID-19 vaccination (Byambasuren et al., 2023; Tran et al., 2023). Unfortunately, data in children are lacking. Vaccines have been found effective in pediatric patients and there are no reasons to think that a positive effect also on the pediatric LC cannot be achieved. However, the true importance of vaccines in this regard is far from be precisely defined. As evidenced in the systematic review by Byambasuren et al. (2023), most of the available studies have significant flaws. Moreover, when prevalence and course of LC was calculated the simultaneous effect of other factors capable of influencing LC prevalence, including role of variants and difference in efficacy of different COVID-19 vaccines was not taken into account (Edwards and Hamilton, 2023).

## 6. Conclusion

Although there is clear evidence that SARS-CoV-2 infection can give rise to post-acute consequences, including LC, determining true prevalence of this condition is very difficult, particularly in children. The complexity in defining and measuring LC is the most important factor that limits present knowledge on pediatric LC. Only recently, a largely accepted definition of this condition in children has been prepared, paving the way for studies with fewer limitations and more reliable results. Although there are already reviews and meta-analysis on the same topic, this narrative review gives a significant contribution in understanding the role of viral variants and vaccines on long COVID development in pediatrics. However, future evaluations of pediatric LC and the factors that can influence its prevalence remain problematic considering that virus variants and vaccines can significantly impact on SARS-CoV-2 circulation and COVID development and severity. Presently available data seem to indicate that most of the long-term clinical manifestations shown in children during pandemic depend on the disproportionate impact on children’s health and well-being of the restrictive measures put in place to contain pandemic. Provisional data regarding the role of variants and vaccines seem to suggest that these factors could have a positive effect contributing to reduce LC prevalence. Unfortunately, data collected in children in these regards are very few or totally inexistent. Moreover, the elimination of restrictive measures makes think that also the problems strictly related to chronic stress could decrease, although presently no clear evidence of this positive effect has been reported. These findings seem to indicate that to face long-term problems of COVID-19 pandemic, together with the use of measures active against SARS-CoV-2 such as vaccines and drugs, initiatives to face psychological problems and chronic stress should be not only maintained but significantly increased. However, to avoid wrong initiatives, a precise evaluation of frequency and clinical characteristics of pediatric LC is needed and a precise differentiation of what happens in children due to the virus and due to the pandemic is urgently needed.

## Author contributions

SE co-wrote the first draft of the manuscript and supervised the project. MD and GR performed the literature review. MP and CC gave a substantial scientific contribution. NP proposed the project and wrote the first draft of the manuscript. All authors contributed to the article and approved the submitted version.

## Funding

The publication of this manuscript was supported by the Laboratory for Clinical Pediatric Research, University of Parma, Parma, Italy (PED-2023-03).

## Conflict of interest

The authors declare that the research was conducted in the absence of any commercial or financial relationships that could be construed as a potential conflict of interest.

## Publisher’s note

All claims expressed in this article are solely those of the authors and do not necessarily represent those of their affiliated organizations, or those of the publisher, the editors and the reviewers. Any product that may be evaluated in this article, or claim that may be made by its manufacturer, is not guaranteed or endorsed by the publisher.
